# Bias in the physical examination of patients with lumbar radiculopathy

**DOI:** 10.1186/1471-2474-11-275

**Published:** 2010-11-30

**Authors:** Pradeep Suri, David J Hunter, Jeffrey N Katz, Ling Li, James Rainville

**Affiliations:** 1 Department of Physical Medicine and Rehabilitation, Harvard Medical School, Boston, MA, USA; 2 Spaulding Rehabilitation Hospital Network, Boston, MA, USA; 3 New England Baptist Hospital, Boston, MA, USA; 4 VA Boston Healthcare System, Boston, MA, USA; 5 Northern Clinical School, The University of Sydney, Sydney, New South Wales, Australia; 6 Division of Rheumatology, Immunology and Allergy, Department of Medicine and Department of Orthopedic Surgery, Brigham and Women's Hospital, Harvard Medical School, Boston, MA, USA

## Abstract

**Background:**

No prior studies have examined systematic bias in the musculoskeletal physical examination. The objective of this study was to assess the effects of bias due to prior knowledge of lumbar spine magnetic resonance imaging findings (MRI) on perceived diagnostic accuracy of the physical examination for lumbar radiculopathy.

**Methods:**

This was a cross-sectional comparison of the performance characteristics of the physical examination with blinding to MRI results (the 'independent group') with performance in the situation where the physical examination was not blinded to MRI results (the 'non-independent group'). The reference standard was the final diagnostic impression of nerve root impingement by the examining physician. Subjects were recruited from a hospital-based outpatient specialty spine clinic. All adults age 18 and older presenting with lower extremity radiating pain of duration ≤ 12 weeks were evaluated for participation. 154 consecutively recruited subjects with lumbar disk herniation confirmed by lumbar spine MRI were included in this study. Sensitivities and specificities with 95% confidence intervals were calculated in the independent and non-independent groups for the four components of the radiculopathy examination: 1) provocative testing, 2) motor strength testing, 3) pinprick sensory testing, and 4) deep tendon reflex testing.

**Results:**

The perceived sensitivity of sensory testing was higher with prior knowledge of MRI results (20% vs. 36%; p = 0.05). Sensitivities and specificities for exam components otherwise showed no statistically significant differences between groups.

**Conclusions:**

Prior knowledge of lumbar MRI results may introduce bias into the pinprick sensory testing component of the physical examination for lumbar radiculopathy. No statistically significant effect of bias was seen for other components of the physical examination. The effect of bias due to prior knowledge of lumbar MRI results should be considered when an isolated sensory deficit on examination is used in medical decision-making. Further studies of bias should include surgical clinic populations and other common diagnoses including shoulder, knee and hip pathology.

## Background

Diagnostic tests are of vital importance in clinical decision-making. In acknowledgment of this fact, guidelines such as the Standards for Reporting of Diagnostic Accuracy (STARD) have been established to improve the quality of design and reporting in diagnostic accuracy studies[1]. The aim of these guidelines is to minimize bias and variation which may affect both the internal and external validity of study results. Nevertheless, few published diagnostic studies meet all methodologic criteria, leaving clinicians with the burden of determining the importance of methodologic shortcomings in published studies, and deciding which study results are most applicable to a given clinical situation[2]. The available literature on diagnostic test bias demonstrates that while some shortcomings in study design result in significant bias, others do not[2-4].

Advanced diagnostic imaging such as magnetic resonance imaging (MRI) is used commonly in modern spine care. In contrast to the situation in primary care, patients frequently present to spine specialists with the results of spine MRI already available at the initial evaluation. The results of prior imaging are often reviewed by the spine specialist prior to the physical examination; this may occur while the history is being obtained, or while the patient is changing into a gown prior to the physical examination. As a consequence of this common practice, the performance of the physical examination in specialty spine care may be influenced by prior knowledge of the results of MR imaging. Given the well-known prevalence of incidental findings on lumbar spine MRI[5,6], prior knowledge of lumbar MRI results therefore introduces the potential for systematic bias in the performance of the physical examination. Since the detection of abnormalities on physical examination may affect the decision to pursue surgery or further diagnostic testing, bias in the physical examination may have substantial implications for the practice of spine care. The effects of prior knowledge of lumbar spine MRI results on the performance of the physical examination have not been previously studied.

The purpose of this study was to empirically assess the effects of bias due to prior knowledge of spine MRI on the perceived diagnostic accuracy of the physical examination for lumbar radiculopathy. We utilized data from a prospective cohort study of lumbar disk herniations to compare the performance characteristics of the physical exam in the ideal situation where the physical examination is performed independently of MRI results (the 'independent group'), with performance in the situation where the physical examination is not performed independently of MRI results (the 'non-independent group'), using a reference standard of the final diagnostic impression of nerve root impingement by the examining physician. Our design acknowledges the potential circularity arising from the fact that the physical exam and final diagnostic determination are performed by the same clinician. We examine the extent that prior knowledge of MRI further influences physical exam interpretation. We hypothesized that estimates of physical exam sensitivity and specificity would be overestimated in the absence of proper blinding to spine MRI. The rationale behind this hypothesis was that foreknowledge of a positive MRI finding might bias towards increased sensitivity by leading to a more focused examination in areas of suspected anatomic pathology. Similarly, foreknowledge of a negative MRI finding might bias towards increased specificity by leading to a less focused examination or a null interpretation of equivocal findings in areas where MRI indicated no anatomic pathology.

## Methods

### Study Participants

**Figure 1:**
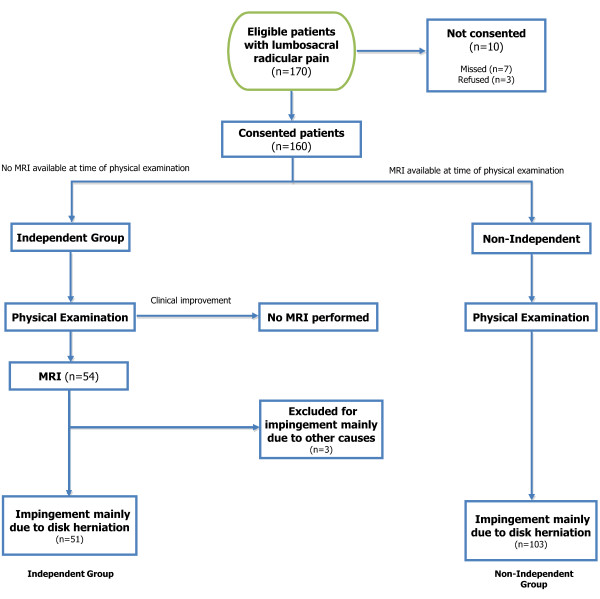
**Flowchart of patient recruitment and participation**.

This was an ancillary study to a prospective evaluation of the outcomes of lumbar disk herniation. The study was approved by the Institutional Review Board of New England Baptist Hospital, Boston. Participants were recruited from a hospital spine center between January 2008 and March 2009. All consecutive patients age 18 and older with lower extremity radiating pain for less than 12 weeks were evaluated for participation. For the purposes of this study, participants were allocated to two groups according to whether or not they had lumbar spine MRI available to the examining physician at the time of physical examination: the 'independent group' had no MRI results available, and the 'non-independent group' had available MRI results. Inclusion criteria for both groups were the historical features of radicular pain in an L2, L3, L4, L5, or S1 dermatome, with or without neurological symptoms, with a concordant MRI finding of nerve root impingement due primarily to lumbar disk herniation. Exclusion criteria were known pregnancy; severe active medical or psychiatric comorbidities that would limit study participation; the presence of significant central or neuroforaminal stenosis from reasons other than lumbar disk herniation as the likely cause of radicular pain; infectious, inflammatory, or neoplastic cause of radiculopathy; significant degenerative or isthmic spondylolisthesis suspected of contributing to symptoms; prior lumbar spine surgery at the affected level. With patients who had no MR imaging available (independent group), it was not possible to confirm whether impingement due to LDH was present at the baseline evaluation. For practical reasons, these patients were offered informed consent at the baseline evaluation, but did not contribute information to the analyses presented here unless their subsequent MRI imaging met study criteria (Figure 1).

### Physical Examination

Participants in both the independent and non-independent groups received a standard battery of physical examination tests which are used commonly in specialty spine care, and are routinely administered in a stereotyped manner in our clinic for the evaluation of lumbosacral radicular pain. Table 1 summarizes the physical examination tests performed; details of the testing methods used in this study are described in depth elsewhere [7-11]. The physical examination consisted of four components: 1) provocative testing, 2) motor strength testing, 3) pinprick sensation testing, and 4) deep tendon reflex testing. Although manual muscle testing (MMT) is most commonly used for the grading of motor strength, we substituted two functional tests of strength in lieu of MMT: the heel-raise test for detection of S1 involvement, and the sit-to-stand test for detection of L3 involvement; the performance characteristics of the latter test have been reported elsewhere. Each participant was examined by one of six board-certified physiatrists specializing in spine care. All physical examination tests were performed bilaterally. Testing results were documented by the examiner in reference to the symptomatic limb; for example, a positive SLR was documented if positive for reproduction of radicular pain in the symptomatic limb. In a minority of cases, where bilateral symptoms existed, the results of testing were documented in reference to the limb that was most painful. The examining physician prospectively recorded information on demographics, historical features, and physical examination findings for all participants using a standardized data sheet.

**Table 1:** Descriptions of Physical Examination Tests and Involved Nerve Roots

Physical Examination Test	Description Of Test	Involved Nerve Roots
**1. Provocative testing**		
Straight Leg Raise (SLR)	With the patient supine, the examiner grasps the patient's heel on the symptomatic (ipsilateral) side while maintaining the knee extended. The straight leg is slowly raised until pain occurs; reproduction of radicular pain constitutes a positive test^1^.	L5, S1
Crossed Straight Leg Raise (CSLR)	The straight leg raise test is performed as above, but is performed instead on the patient's well leg. Reproduction of radicular pain in the symptomatic limb constitutes a positive test^1^.	L5, S1
Femoral Stretch Test (FST)	With the patient prone, the examiner grasps the patient's ankle on the symptomatic (ipsilateral) side and facilitates ipsilateral knee flexion; reproduction of typical lower extremity pain constitutes a positive test.	L2, L3, L4
Crossed Femoral Stretch Test (CFST)	With the patient prone, the examiner grasps the patient's ankle on the asymptomatic (contralateral) side and facilitates contralateral knee flexion; reproduction of typical lower extremity pain constitutes a positive test.	L2, L3, L4
**2. Motor testing**		
Hip flexion test	The patient lies supine and flexes the ipsilateral hip while the examiner applies an extension force; inability to resist examiner is a positive test result.	L2
Sit-to-stand test	The test begins with the patient sitting and the examiner standing facing the patient. The patient rises to standing using only the strength of one supporting limb, holding the examiner's hands for balance; inability to do so is a positive test^2^.	L3
Heel walk test	The patient walks on heels only while avoiding contacting the floor with the forefoot, using the examiner's for balance as needed; inability to maintain the forefoot off the ground is a positive result.	L4
Great toe extensor strength	The patient fully dorsiflexes the great toe and maintains this position as the examiner applies a plantarflexion force; inability to do so is a positive result.	L5
Heel raise test	The patient stands on one foot while flexing the contralateral knee, and then plantarflexes the ankle, raising the heel of the supporting limb off the floor to maximal plantarflexion. Inability to perform 10 successive heel raises is a positive result.	S1
**3. Sensory testing**		
Anterior thigh sensation	Sensation is assessed by pinprick testing at the mid-anterior thigh using a standard 3 point grading scale^3^; any sensory impairment is a positive result.	L2
Medial knee sensation	Sensation is assessed by pinprick testing at the medial aspect of the knee; any sensory impairment is a positive result.	L3
Medial ankle sensation	Sensation is assessed by pinprick testing at the medial aspect of the ankle; any sensory impairment is a positive result.	L4
Great toe sensation	Sensation is assessed by pinprick testing at the dorsal aspect of the great toe; any sensory impairment is a positive result.	L5
Lateral foot sensation	Sensation is assessed by pinprick testing at the lateral border of the foot; any sensory impairment is a positive result.	S1
**4. Reflex testing**		
Patella reflex	Achilles deep tendon reflex is assessed using a standard 5 point grading scale^4^; diminished grade as compared to the contralateral limb is a positive result.	L4
Achilles reflex	Patellar deep tendon reflex is assessed and graded as above; diminished grade as compared to the contralateral limb is a positive result.	S1

### Correlation of Physical Examination Tests to Lumbosacral Nerve Root Level

The physical examination for lumbar radiculopathy is important not only for the identification of whether radiculopathy is present, but for anatomic localization of radiculopathy. Specific physical examination tests are therefore conceptually most appropriate for the detection of specific pathology. For example, the straight leg raise test is most clinically applicable for the detection of nerve root pathology at either the L5 or S1 levels (low lumbar impingement)[12], while the femoral stretch test is most applicable for the detection of nerve root pathology at the L2, L3, or L4 levels (midlumbar impingement)[13]. On the other hand, some tests are most applicable for the detection of level-specific nerve root involvement, such as in the case of Achilles reflex testing for S1 pathology[14]. Although various classification systems exist for relationships between physical examination tests and the localization of level-specific nerve root dysfunction, the American Spinal Injury Association (ASIA) classification for sensory and motor testing at the L2-S1 levels is commonly used by spine physiatrists[14]. Table 1 summarizes the relationships between individual physical examination tests and the specific nerve root levels or combinations of levels they are intended to test, and as utilized in our analytic approach. The system of classification as presented in Table 1 is consistent with the ASIA classification[14], textbooks of neurophysiology[15], and is reflective of standard practice in our clinic.

### Magnetic Resonance Imaging Studies

All patients received MRI imaging of the lumbar spine, which consisted at minimum of T1 and T2 weighted images in the sagittal and axial planes. Participants in the independent group did not have spine MRI available to the examining physician at the time of their physical examination, and therefore the examination was blinded to MRI results. These patients went on to receive lumbar spine MRI according to usual practice in our clinic[16]. The decision to obtain MRI is a clinical determination based on general criteria of diagnostic evaluation for symptoms of sciatica of approximately 6 weeks in duration[16]. In cases of severe pain or neurologic progression, MR may be obtained substantially earlier than 6 weeks. Participants in the non-independent group presented with the results of lumbar spine MRI available at the time of their physical examination, and therefore the examination was not blinded to MRI results. It is usual practice in our clinic to review available MRI results while the patient is changing into a gown, prior to the physical examination.

### Classification of Nerve Root Impingement

The final diagnostic impression of the symptomatic level of nerve root impingement by the examining physician, as recorded on the standardized data collection sheet, was used as the reference standard for this study. This composite reference standard reflects the overall diagnostic impression of the examining physician, taking into account the results of the clinical evaluation, the physician interpretation of spine MRI, and the radiologist interpretation of spine MRI. MRI results were therefore incorporated into the composite reference standard for final physician diagnostic impression for both the dependent and independent groups. As such, this composite reference standard accurately reflects the process of diagnosis in standard clinical practice. In situations where nerve root impingement at more than one level was possible, the level thought to be primarily responsible for the production of symptoms was chosen as the reference standard.

### Statistical Analysis

To characterize the demographics, clinical characteristics, and radiographic features of the independent and non-independent groups, we calculated means and standard deviations for continuous variables, and frequencies and proportions for categorical variables. Our analytic approach was based on a comparison of test performance characteristics in the independent group (with blinding to spine MRI) and the non-independent group (without blinding to spine MRI), using a reference standard of the final classification of lumbar nerve root impingement by the examining physician. For analytic purposes, we conducted separate analyses for each of the four physical examination components (provocative testing, motor strength testing, pinprick sensation testing, and deep tendon reflex testing). Table 1 summarizes the relationships between individual physical examination tests and specific nerve root levels or combinations of levels employed in this analysis. We constructed two-by-two contingency tables for each examination component in the independent and non-independent groups separately. Sensory testing and motor testing contingency tables were populated with the results of testing at the individual nerve root level, rather than the results of testing at the subject level. For example, in the construction of the sensory testing contingency table, each subject contributed the results of pinprick sensory testing at each individual sensory level from L2 to S1, for a total of five sensory levels per subject. For provocative testing, each subject contributed the results of straight leg raise testing and crossed straight leg raise testing for the low lumbar levels (L5 or S1), and femoral stretch testing and crossed femoral stretch testing for the midlumbar levels (L2, L3, or L4) to the contingency table. For reflex testing, each subject contributed the results of patellar tendon reflex testing (L4) and Achilles tendon reflex testing (S1) to the contingency table. In this manner, each study subject contributed 'case' information from their symptomatic level of nerve root impingement, as well as 'control' information from non-affected nerve root levels. For example, for motor strength testing, a subject with L3 nerve root impingement contributed 'case' information based on the L3 level, but also contributed 'control' information based on the L2, L4, L5, and S1 levels. We then calculated sensitivities and specificities, including 95% confidence intervals (CIs), for each test component in both the independent and non-independent groups[17]. We compared estimates of sensitivity and specificity between the independent and non-independent groups using the chi-square test. All analyses were performed using SAS software, version 9.0 (SAS Institute., Cary, NC).

## Results

Participant recruitment for this study is depicted in Figure 1. Of 170 potential participants, 10 individuals either declined to participate or were missed by the recruiting physicians. 160 participants were consented, including 57 participants who had no imaging available at baseline, and 103 participants who had an available lumbar MRI with evidence of nerve root impingement due to lumbar disk herniation. The 103 participants with available MRI constituted the non-independent group. Of the 57 participants with no imaging available at baseline, three participants did not go on to receive MRI due to clinical improvement, and were excluded from this analysis. 54 participants who had no imaging available at baseline went on to receive MRI, though three additional participants were subsequently excluded for having impingement not primarily due to lumbar disk herniation, leaving 51 participants in the independent group.

Demographics and clinical characteristics of the study sample are presented in Table 2. Average age, leg pain, back pain, and comorbidity were comparable between the independent and non-independent groups. There were fewer females (21.6% vs. 37.9%; p = 0.04) and shorter duration of symptoms (4.3 vs. 5.2; p = 0.08) in the independent group. Oswestry Disability Index (ODI) scores showed less impairment in the independent group than in the non-independent group. (45 vs. 54; p = 0.014). Pain intensity for leg pain and back pain were comparable between groups.

**Table 2:** Characteristics of Independent vs. Non-independent Groups

Characteristic	**Independent Group (n = 51)**^**+**^	**Non-independent Group (n = 103)**^**+**^	
Age (yrs)	53.9 (15.0)	52.6 (12.9)	p = 0.56
Female (%)	11 (21.6%)	39 (37.9%)	p = 0.04*
Katz Comorbidity Score (0-45)	2.7 (3.3)	2.9 (3.3)	p = 0.70
Symptom duration (wks)	4.3 (2.8)	5.2 (3.1)	p = 0.08
Oswestry Disability index (0-100)	45 (20)	54 (21)	p = 0.014*
VAS Leg Pain (0-10)	7.1 (2.5)	6.9 (2.4)	p = 0.60
VAS Back Pain (0-10)	5.1 (3.3)	5.1 (3.3)	p = 0.94
Midlumbar nerve root impingement (L2, L3, or L4 levels)	24 (47.1%)	54 (52.4%)	p = 0.53

*statistically significant

^+^Mean (S.D.) or N (%)

**Figure 2:**
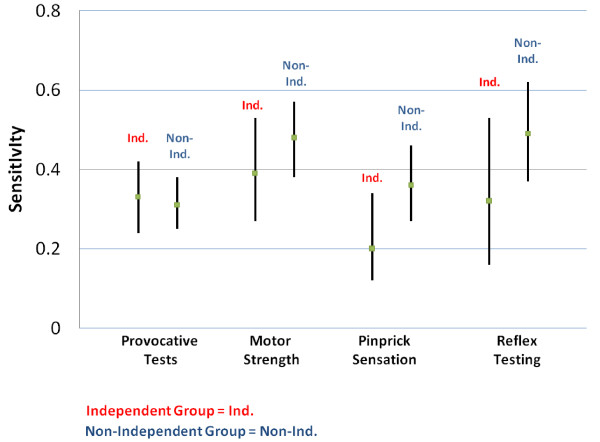
**Comparison of Perceived Sensitivity with Physician Blinding (Independent) and without Physician Blinding (Non-Independent) to MRI**.

The performance characteristics of provocative testing, motor testing, sensory testing, and reflex testing for the diagnosis of lumbar radiculopathy are presented in Table 3. The perceived sensitivity of pinprick sensory testing was higher with prior knowledge of MRI results than without (36% vs. 20%; p = 0.05). The perceived sensitivity of deep tendon reflex testing was higher with prior knowledge of MRI results than without, but this was not statistically significant (49% vs. 32%; p = 0.17). Sensitivities and specificities for the exam components of provocative testing, motor testing, sensory testing, and reflex testing otherwise also showed no significant differences between groups. Figure 2 presents a graphical illustration of point estimates and 95% confidence intervals for the perceived sensitivity of different components of the physical examination. A tendency towards a higher perceived sensitivity is noted with respect to pinprick sensation and reflex testing.

**Table 3:** Comparison of the Performance Characteristics of Physical Examination Tests with Physician Blinding to Imaging (Independent), and No Physician Blinding to Imaging (Non-Independent)

Examination Component	Independent Group	Non-Independent Group	p-value (between groups)
	Sens. (95% CI)	Sens. (95% CI)	
**Sensitivity**			

**Provocative testing**	33 (24-42)	31 (25-38)	0.77
**Motor strength testing**	39 (27-53)	48 (38-57)	0.33
**Pinprick sensory testing**	20 (12-34)	36 (27-46)	0.05
**Reflex testing**	32 (16-53)	49 (37-62)	0.17

**Specificity**			

**Provocative testing**	95 (89-98)	94 (89-96)	0.66
**Motor strength testing**	89 (84-92)	86 (82-89)	0.35
**Pinprick sensory testing**	93 (89-96)	92 (89-94)	0.49
**Reflex testing**	90 (81-95)	92 (86-95)	0.59

**Sens**. - Sensitivity (%); **Spec**. - Specificity (%)**;CI **- Confidence interval

## Discussion

The primary finding of this study is that prior knowledge of lumbar MRI results may have the potential to introduce bias into the pinprick sensory testing component of the physical examination for lumbar radiculopathy, by increasing the perceived sensitivity of sensory testing. No statistically significant effect of bias was seen for deep tendon reflex testing, motor strength testing or provocative maneuvers. This finding suggests that bias due to prior knowledge of MRI results should be considered when abnormal results on sensory testing are the only deficit noted on physical examination, and when this information is used for medical decision-making.

The bias introduced to the physical examination by prior knowledge of lumbar MRI is a result of many factors, but appears similar in form to clinical review bias. Clinical review bias occurs when the availability of clinical information- or in this case, imaging results- during interpretation of the index test affects the final diagnosis[2]. Although we are aware of no prior studies examining the effects of bias in the musculoskeletal physical examination, our findings are consistent with prior investigations of clinical review bias from the radiology literature, which have demonstrated increases in sensitivity when clinical information is available during test interpretation[18-20]. The reported effects of clinical review bias on test specificity have ranged from small increases[21], to no change[18], to reductions[20]. Our finding of bias in sensory testing- but not in other components of the examination- is consistent with prior observations that the potential for bias increases with increasing subjectivity in the interpretation of the index test[22]. In the current study, provocative maneuvers which rely on patient self-report of typical pain reproduction, and motor testing using functional tests of resistance applied against the patient's own body weight, may have resulted in more objective interpretation, which was less susceptible to bias. It should be noted that for the reflex examination, where there can be much subjectivity in ascertaining subtle side-to-side differences in testing, there were differences in estimates of sensitivity that suggested bias due to foreknowledge of MRI results, although these did not reach the threshold of statistical significance. The need for greater understanding of the bias produced by physician knowledge of imaging results is underscored by health services studies[23] and clinical trials[24], which have found associations between increased availability of MR imaging and higher rates of spine surgery.

The observed effect of bias on the sensory and reflex testing components of the physical examination draws attention to subtleties of the radiculopathy exam. The term '*perception*' is used in diagnostic testing to refer to the process of identification of abnormal areas[25]. Prior knowledge of MRI results in our study may have altered physician perception, either by lowering the threshold of abnormality when MRI suggested nerve impingement at a specific spinal level, or raising the threshold of abnormality when MRI appeared normal. Prior knowledge of MRI results may also alter physician perception by focusing attention on the results of specific tests, while decreasing attention paid to other tests. An important unanswered question is whether the results of physical examination are more valid or less valid with blinding to MRI results. Although formal guidelines for study design would suggest greater validity in interpretation of the physical exam with blinding to MRI results, it remains to be seen if such blinding results in improved accuracy using a reference standard that incorporates clinical outcomes. Further studies of physical examination bias are needed to determine the true effect of prior knowledge of MRI results on diagnostic accuracy. These studies should include surgical clinics, where abnormalities in the physical exam may have immediate implications for surgical decision-making, and should examine other common diagnoses in musculoskeletal medicine including shoulder, knee and hip pathology.

This study has several limitations. First, our use of the composite reference standard of final clinician diagnosis (combining clinical impression and MRI assessment into a final diagnostic impression) may be perceived as imperfect. We believe that the composite reference standard used in this study is appropriate, in that it reflects the process of diagnosis used by physicians in actual clinical practice. Second, elements of incorporation bias (where the result of the index test is used to establish the final diagnosis), and test-review bias (where there is inadequate blinding of the person interpreting the index test to the reference standard) may have come into play with this study design[2,4]. Although these limitations exist, the aforementioned biases would be expected to affect both independent and non-independent groups equally. A prior systematic review, moreover, found no significant effect of bias due to a composite reference standard or incorporation bias [4]. Although the fact that some individuals (3) in the independent group did not go on to receive imaging due to clinical improvement may have introduced some differential bias, we would expect this bias to be quite small given the number of individuals involved. Third, in general, aspects of the design of this study may have oversimplified situations which are more complicated in actual practice. For example, only individuals with radicular pain and MRI evidence of nerve root impingement due primarily to disk herniation were included in the study, and the final clinician diagnosis required the attribution of symptoms to a single nerve root. Although these factors also would be expected to affect both groups equally, they may have overestimated accuracy or introduced variability, which could obscure the bias conferred by prior knowledge of imaging results. The summary performance characteristics presented here should be viewed in this context; these estimates pertain to the localization of nerve root impingement in a selected population, and should not be compared to those yielded by prior studies of the physical examination for the identification of lumbar disk herniation[26]. Future studies may also consider investigating the effects of bias outside the setting of a structured research protocol, where 'real world' practice may greatly increase the effect of bias due to prior knowledge of MRI results.

## Conclusions

The physical examination is arguably the most commonly employed diagnostic test in musculoskeletal medicine, and possesses the advantages of incurring relatively low cost and low patient risk. Nevertheless, to our knowledge, this study is the first to evaluate the effects of systematic bias in the musculoskeletal physical examination. Prior knowledge of lumbar MRI results may introduce bias into the sensory testing components of the physical examination for lumbar radiculopathy. The effects of this bias should be considered when an isolated sensory deficit on examination is used in medical decision-making. Further studies of bias in other aspects of the musculoskeletal physical examination are warranted.

## Competing interests

The authors declare that they have no competing interests.

## Authors' contributions

PS was involved with study concept and design, acquisition of data, analysis of data, interpretation of data, and drafting of the manuscript. DJH was involved with study concept and design, analysis of data, interpretation of data, and manuscript preparation. JNK was involved with study design, analysis of data, interpretation of data, and manuscript preparation. LL was involved with analysis of data, interpretation of data, and manuscript preparation. JR was involved with study concept and design, acquisition of data, interpretation of data, and manuscript preparation. All authors were involved with critical revision of the manuscript for important intellectual content and approved the final version of the manuscript.

## Pre-publication history

The pre-publication history for this paper can be accessed here:

http://www.biomedcentral.com/1471-2474/11/275/prepub
